# Application of sedation–agitation scale in conscious sedation before bronchoscopy in children

**DOI:** 10.1097/MD.0000000000014035

**Published:** 2019-01-04

**Authors:** Lin Zhong, Kun Shen, Songhui Zhai, Ting Chen, Qingfen Tao, Lina Chen, Yuhong Tao, Li Qiu

**Affiliations:** a Department of Pediatric Pulmonology and Immunology, West China Second University Hospital of Sichuan University; b Key Laboratory of Birth Defects and Related Diseases of Women and Children (Sichuan University), Ministry of Education; c Department of Orthopedics, Nuclear Industry 416 Hospital; d The Second Affiliated Hospital of Chengdu Medical college, Chengdu, Sichuan, China.

**Keywords:** bronchoscopy, children, conscious sedation, midazolam, sedation–agitation scale

## Abstract

This retrospective study investigated the application of the sedation–agitation scale (SAS) in pediatric bronchoscopy by observing its effects on sedative dosages and adverse reactions.

Children who underwent sedation before bronchoscopy, during the period from January 2014 to June 2017, were divided into control and SAS groups. Patients in the control group were administered a single dose of 0.1 to 0.3 mg/kg midazolam, based on physicians’ clinical experience. The initial dose of midazolam in the SAS group was 0.1 mg/kg, and was adjusted based on the SAS score, as evaluated by physicians. Between-group comparisons were made of midazolam dose; adverse reactions of midazolam, such as agitation, delirium, excessive sedation, and respiratory depression; operating time of bronchoscopy; and number of participants.

No statistically significant differences in gender, age distribution, weight, or disease composition were observed between the groups. The midazolam dose, operating time, and number of participants at different ages were all lower in the SAS group than in the control group. Fewer adverse drug reactions, such as intraoperative agitation and delirium, were noted in the SAS group. Moreover, the overall number of participants was reduced, and the overall operating time was less than that in the control group.

Application of SAS for assessment of sedation during pediatric bronchoscopy can guide individualized administration of midazolam, reduce midazolam dose while achieving an ideal sedative effect, reduce adverse reactions, and improve operator experience. Hence, its use should be promoted for pediatric patients undergoing bronchoscopy under local anesthesia and conscious sedation.

## 1 Introduction

Sedation or anesthesia is an indispensable step in pediatric bronchoscopy. Currently, anesthesia methods in bronchoscopy include surface anesthesia of the mucous membrane (after conscious sedation) and general anesthesia. General anesthesia has various disadvantages, including respiratory depression, increased difficulty in intraoperative and postoperative respiratory management, requirements for anesthesiologist involvement and use of operating suites, slow postoperative patient recovery, and high cost.^[1–3]^ Therefore, conscious sedation and surface anesthesia of the local mucous membrane are typically used for pediatric patients with stable disease conditions who require simple examinations with short operating time, and for those in whom dynamic changes in airways and vocal cords must be observed.^[4]^ This can avoid the side effects of general anesthesia and simplify respiratory management. Furthermore, it is unnecessary to use an operating suite, and the involvement of anesthesiologists is not required. However, questions remain regarding which medications should be used for conscious sedation of children, what methods and standards are appropriate to evaluate the level of sedation in children, and how to optimally reduce the occurrence of adverse drug reactions while ensuring a smooth operation.

Midazolam is a preferred medication for conscious sedation due to its rapid onset, short duration of action, wide range of safety, and inability of patients to recall events that occurred during sedation. It is recommended by the “Guideline of Pediatric Bronchoscopy (2009 Edition)” by the Pediatrics Branch of the Chinese Medical Association^[5]^ and the “Sedation Guideline for Children and Adolescents Care Practice” by the British NICE (the National Institute for Health and Care Excellence) (2010)^[6,7]^; these specify that midazolam should be considered for conscious sedation during bronchoscopy in children and adolescents. However, some children experience delirium, agitation, and aggressive behaviors after administration of midazolam, affecting bronchoscopy or requiring excessive sedation that requires long-term supervision. Hence, the individualized application of midazolam has been suggested. The use of appropriate assessment tools during sedation facilitates the measurement of sedative level, facilitates an uncomplicated operation, and reduces drug side effects in children. The Riker Sedation–Agitation Scale (SAS)^[8]^ is a tool for the subjective assessment of sedation status. Notably, it is simple to operate, and its feasibility and validity are equivalent to those of objective assessments. SAS has been recommended for the evaluation of sedative levels in children by expert consensus regarding sedation treatment in the pediatric intensive care unit (ICU). In the present study, the SAS score^[8]^ was used to guide the application of sedatives for children undergoing bronchoscopy who needed preoperative sedation, in order to assess the clinical application of the SAS in preoperative sedation for pediatric bronchoscopy.

## 2 Subjects and methods

### 2.1 Study participants

Study participants were hospitalized children undergoing routine bronchoscopy and bronchoalveolar lavage at the West China Second Hospital of Sichuan University, during the period from January 2014 to June 2017. The exclusion criteria were as follows: infants younger than 1 year; patients with severe hepatorenal insufficiency, heart failure, or respiratory failure who could not tolerate bronchoscopy; patients with severe upper airway obstruction, for whom sedation may induce aggravation of obstruction or cause deterioration in disease conditions; patients with mechanical ventilation; patients requiring bronchial biopsy or transbronchial lung biopsy; and patients for whom bronchoscopy was performed under general anesthesia in one of the following conditions: tracheobronchial aspiration or endogenous foreign bodies, airway stenosis requiring balloon inflation or thermal ablation, life-threatening hemoptysis, voluntary selection by patients or their guardians. This was a retrospective controlled study. Pediatric patients between January 2014 and December 2015, who were not evaluated by SAS, were placed in the control group; pediatric patients from January 2016 to June 2017, who were evaluated by SAS, were included in the SAS group. Midazolam dosage and its adverse reactions, such as agitation, delirium, and respiratory depression, were compared between the 2 groups. Moreover, operation characteristics, such as operating time of bronchoscopy and the number of participants, were also compared. Before the operation, guardians of all patients provided informed consent for both bronchoscopy and preoperative sedation, together with anesthesia. This study was approved by the Ethics Committee of West China Second Hospital of Sichuan University.

### 2.2 Anesthesia

Surface anesthesia for the nasal cavity and throat was performed by using 2% lidocaine, 10 to 15 minutes before the operation, for all pediatric patients. After the BF-P260/BF-XP260 bronchoscope (Olympus, Japan) entered the trachea, 1 to 2 mL of 2% lidocaine was added for children more than 1 year of age. Subsequently, 1 to 2 mL of 1% lidocaine was added after the bronchoscope entered the left and right main bronchi. The total dose of lidocaine did not exceed 5 to 7 mg/kg throughout the procedure.

### 2.3 Conscious sedation guided by the SAS score

Standards for SAS scoring are^[8,9]^ listed in Table 1.

**Table 1:**
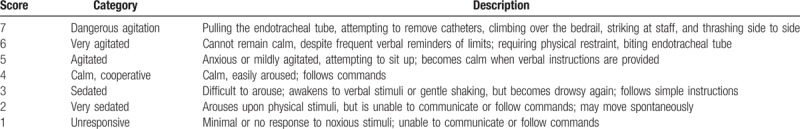
Standards of sedation–agitation scale scoring.

**Figure 1:**
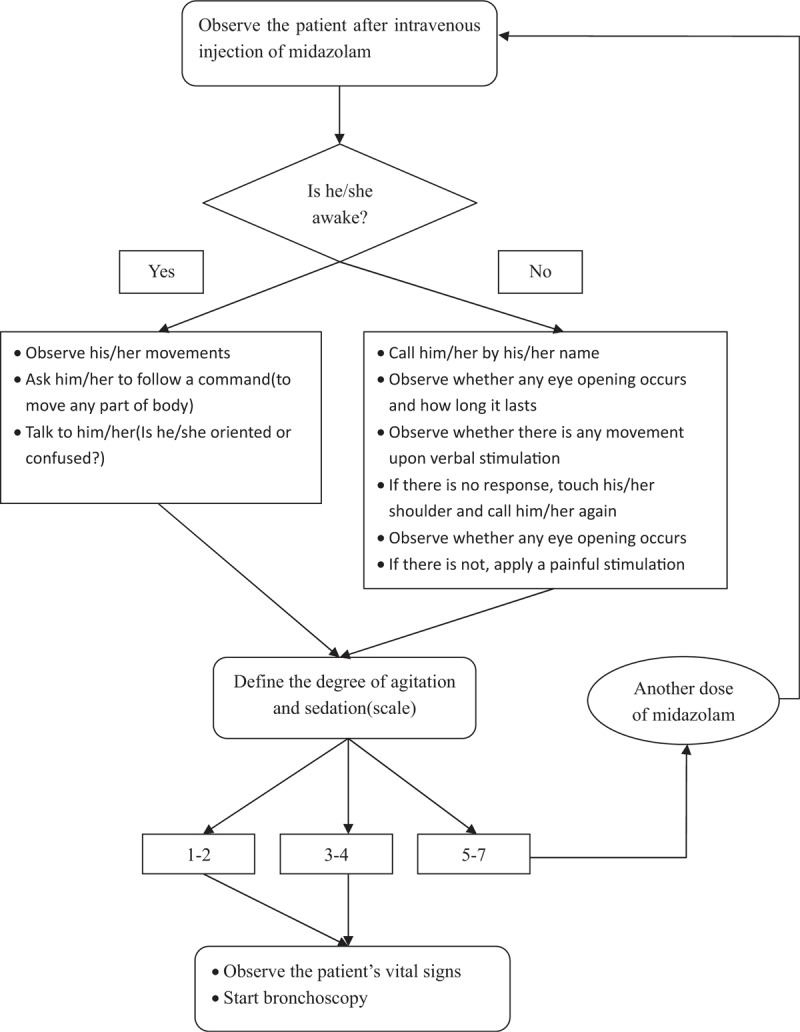
Study protocol for evaluating sedation-agitation levels. Operators evaluated patients’ sedation–agitation levels according to the flow in the diagram after the medication of midazolam. If the sedation–agitation scale reached 3 to 4 points or 1 to 2 points, the bronchoscopy could be performed. If the sedation–agitation scale was higher than 4 points, supplemental administration of midazolam was needed and evaluation repeated until the score reached 3 to 4 points.

Conscious sedation was performed 10 minutes before the operation for all pediatric patients. In the control group, SAS scoring was not performed; a single dose of 0.1 to 0.3 mg/kg midazolam was administered on the basis of the physicians’ clinical experience. The administration rate was 1 mg/min; the dose was no >0.3 mg/kg or 10 mg. In the SAS group, conscious sedation was performed by the physicians under the guidance of SAS scoring. Before bronchoscopy, 0.1 mg/kg midazolam was intravenously infused slowly, at a rate of 1 mg/min. SAS was immediately scored by the physicians and nurses. If the sedative effect was not satisfactory, 0.05 mg/kg midazolam was rapidly added and SAS was scored immediately after the infusion. Midazolam was added until the SAS score was 3 to 4 points. The total dose of midazolam did not exceed 0.3 mg/kg or 10 mg. The protocol used for evaluating sedation–agitation is shown in Fig. 1. The same physicians performed operations for both groups. Physicians and nurses participating in the assessment received training for the scoring standard.

### 2.4 Observation outcomes

The observation outcomes were midazolam dose; adverse drug reactions, such as delirium, agitation, respiratory depression; operating time of bronchoscopy; and number of participants. The number of participants referred to the total number of staff participating in the examination, including physicians, nurses, and staff, who assisted in procedures involving the pediatric patients.

### 2.5 Statistical analysis

SPSS20.0 statistical software (IBM, Armonk, NY) was used in the analysis. Continuous data were expressed as mean ± standard deviation 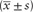. Comparisons of mean values between the 2 groups were performed by using the *t* test. Mean values among multiple groups were compared by using multivariate analysis of variance. Categorical data were expressed as cases and percentage. Group-wise comparisons were performed by using the chi-squared test. A *P* value <.05 was considered statistically significant.

## 3 Results

### 3.1 General data

A total of 422 pediatric patients, including 242 boys and 180 girls, were enrolled. The average age was 5.42 ± 3.84 years (1–17 years). The average weight was 20.08 ± 10.42 kg (5–56 kg). The SAS and control groups comprised 218 and 204 patients, respectively. No statistically significant differences were observed in gender, age distribution, weight, or disease composition between the 2 groups (Table 2).

**Table 2:**
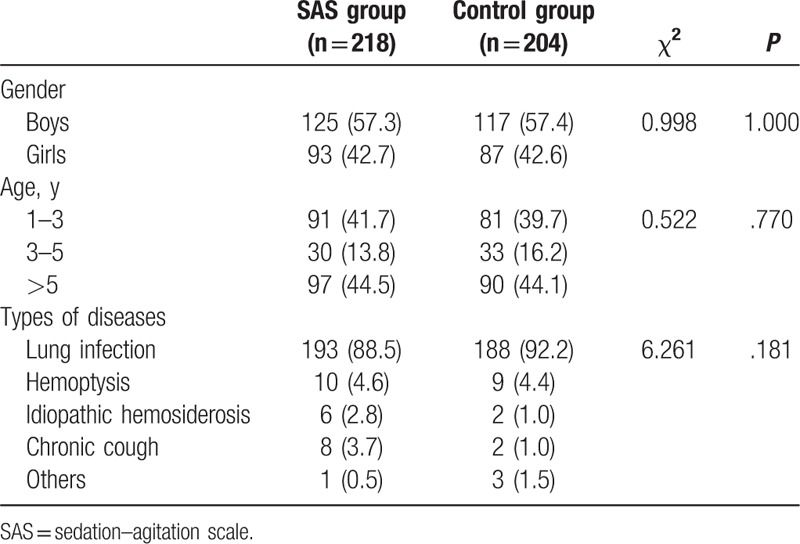
General characteristics of the pediatric patients.

### 3.2 Assessment of SAS-guided sedative effects

The average doses of midazolam were 0.26 ± 0.51 and 0.22 ± 0.06 mg/kg in the control and SAS groups, respectively. The dose was significantly lower in the SAS group than in the control group (*P* < .05) (Table 3). The operating time of bronchoscopy was shorter and the number of participants was reduced in the SAS group. The differences were statistically significant.

**Table 3:**
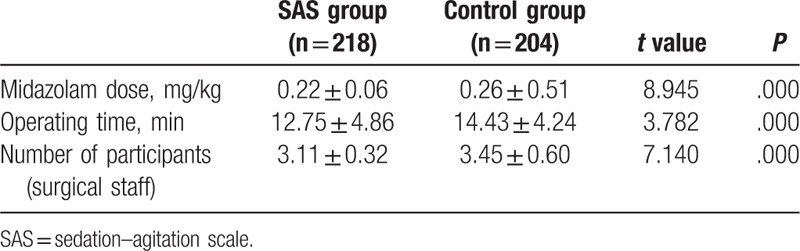
Assessment table of SAS-guided sedative effects.

Further comparisons were performed after stratification of the pediatric patients according to age. Midazolam dose, operating time of bronchoscopy, and number of participants were all significantly lower in the SAS group than in the control group (Table 4). Age also significantly impacted the sedative effects.

**Table 4:**
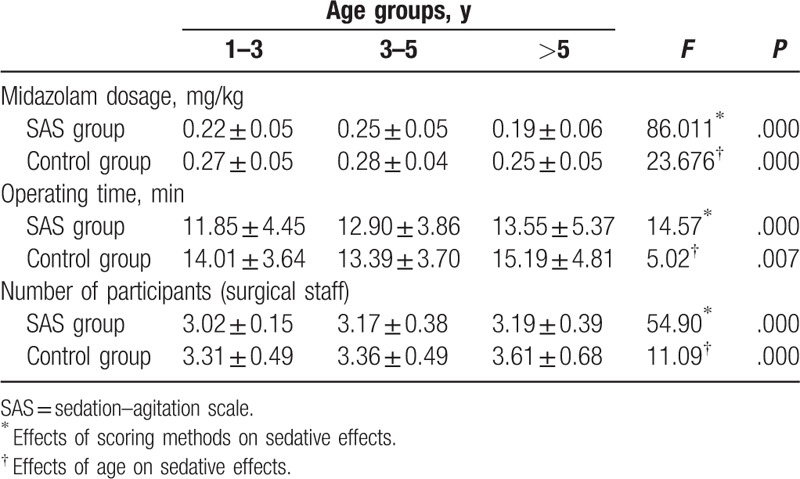
Assessment table of SAS-guided sedative effects at different ages.

### 3.3 Adverse reactions caused by midazolam

All pediatric patients had no abnormalities, such as bucking or aspiration, during the restoration of eating and drinking, 3 hours after the procedures. After intravenous injection of midazolam, most patients could achieve an ideal level of sedation; however, a small number of patients developed delirium and agitation, and a few patients experienced respiratory depression. Of these, 25 patients in the control group and 3 in the SAS group showed significant delirium or agitation (χ^2^ = 20.133, *P* < .05); these differences were statistically significant (Table 5). Many of the pediatric patients with significant agitation were older than 5 years of age. Two patients in the control group experienced respiratory depression; they were all in the 1- to 3-year-old group. Moreover, all patients successfully recovered after symptomatic treatment without adverse consequences. No respiratory depression occurred in the SAS group.

**Table 5:**
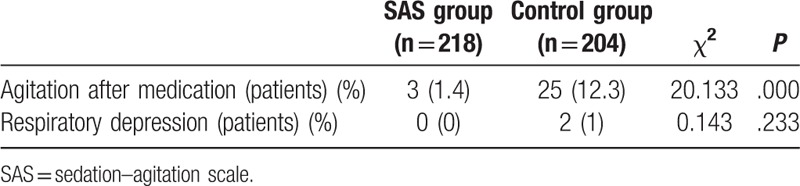
Adverse reactions of midazolam.

## 4 Discussion

The sedation assessment tool has become a necessary means to evaluate the level of sedation in ICUs and surgical operations^[10–12]^; this includes both subjective and objective assessments. Objective assessment is accurate, but requires specialized equipment; moreover, the indicators are numerous and complex. Thus, this approach is limited in clinical application. By contrast, subjective assessment is uncomplicated. Moreover, the feasibility and validity aspects of some assessment methods are comparable to those of an objective assessment. Thus, these are more extensively applied in the clinical setting. Currently, the credibility and applicability of the following 5 commonly used scoring tools^[13]^ have been fully validated and evaluated in adults: Glasgow Coma Scale (GCS), Ramsay Scale, SAS, Motor Activity Assessment Scale (MAAS),^[14]^ and Richmond Agitation–Sedation Scale (RASS).^[15]^ Of these, the Ramsay Scale is most frequently applied in adult bronchoscopy.^[16,17]^ However, the Ramsay Scale evaluates the depth of sedation only with regard to the response intensity of patients to stimuli. Thus, it is of limited use in children. The GCS, MAAS, and RASS involve additional scoring items and their methods are complex. Thus, they are not appropriate for pediatric bronchoscopy. The SAS was proposed by Riker et al in the 1990s^[8,9]^; it was first used for the assessment of sedation and agitation status of severely ill adult patients in the ICU. The SAS obtained the highest psychological score, when evaluating the effects of surgical anesthesia with various assessment tools. The credibility of its participants (including ICU physicians) and judgment credibility were both highest among other methods, such that it could distinguish levels of sedation in different clinical situations. Its score moderately to highly correlated with those of objective assessment tools, including the bispectral index (BIS) and electroencephalogram. The SAS is recommended for use in evaluating the level of sedation in children by an expert consensus regarding pediatric sedation in the ICU.^[18,19]^ The SAS can objectively describe the agitation status of patients when evaluating sedation status, which is conducive for the prediction of difficulty level and progression of examinations. Moreover, it can predict whether physical restraint should be strengthened on pediatric patients, and whether the number of staff assistants should be increased. Further, its scoring items are more feasible. Therefore, SAS was used in the present study to evaluate the level of sedation in children undergoing bronchoscopy, in order to guide the use of midazolam.

The present study found that the average dose of midazolam was lower in the SAS group than in the control group. The proportions of drug-induced delirium and involuntary movement were significantly lower in the SAS group than in the control group, with no adverse reactions (e.g., respiratory depression). This might be because, for the control group, the dose of midazolam was determined on the basis of the preoperative conditions of the pediatric patients, as well as the clinical experience of the attending physicians. All control patients were given a single dose by a nurse; the administration rate was not strictly controlled, and the sedation status during administration was not evaluated. Although the medication was administered within the recommended dose range, the aforementioned adverse reactions of overdose occurred due to individual differences. Moreover, to ensure a sedative effect, physicians might prefer to use a larger dose, which is more likely to result in an adverse reaction. When SAS was used to evaluate the sedation–agitation level, the midazolam dose was gradually increased; injection was performed slowly to allow physicians to comprehensively evaluate sedation status. Therefore, adverse reactions, such as delirium, involuntary movement, and respiratory depression, caused by rapid infusion or excessive dose, could be avoided; this enabled achievement of individualized medication and implementation of the goal of reasonable sedation. Regarding the operation, the average time required in the SAS group was short. In the SAS group, the number of participants (staff members) was reduced, compared with the control group; this suggests that the level of sedation in pediatric patients undergoing procedures was ideal after the use of the SAS, which was conducive to successful and rapid implementation of procedures. Additionally, after the SAS was applied, the midazolam dose decreased significantly in patients more than 5 years of age, with no significant agitation and delirium; this indicated that the required midazolam dose was low for children in the older age group, and that adverse reactions were more likely to occur when a high dose was used. The midazolam dose was higher in the younger age group, compared with the older age group. Nevertheless, adverse reactions (e.g., respiratory depression) occurred in the control group, which might be associated with the incomplete development of respiratory functions or an overdose of midazolam. After the introduction of SAS, no severe adverse reactions occurred in the younger age group, suggesting that assessment of sedation status by using SAS could avoid the occurrence of severe adverse reactions due to an overdose of midazolam.

The results of the present study suggested that, after the introduction of SAS, children undergoing bronchoscopy with local anesthesia and conscious sedation were more likely to achieve an ideal sedation status. Moreover, the operation was uncomplicated and more successful; the number of participants could be reduced, the procedural efficiency could be improved, and the occurrence of severe adverse reactions, especially respiratory depression, could be reduced.

The present study had certain limitations. There may have been a learning bias in terms of operation time because of the operator's proficiency; a skilled operator may have required less time to complete the same procedure. However, proficiency was not the sole influential factor for the operation; sedation and cooperation in the patients strongly influenced operation time. Notably, it was difficult to complete the operation in an uncooperative, agitated patient, regardless of the level of experience of the operating physician. Thus, we suspect that the learning bias had little influence on the result. Because children younger than 3 years old could not communicate effectively with the surgical staff, some indicators could not be used during SAS scoring; this may have led to inaccurate scores. For children older than 5 years of age, some adverse reactions (e.g., agitation) occurred regardless of the application of SAS. Therefore, well-targeted studies involving more patients, and a multicenter prospective study, are needed. It may be effective to compare the effects of different scoring systems, such as BIS or RASS, with that of the SAS. Importantly, the SAS remains applicable for most children.

In summary, the SAS can be used to evaluate the sedation status of pediatric patients, in order to guide sedative dosage, when conscious sedation is performed for children undergoing bronchoscopy with local anesthesia. The occurrence of adverse reactions caused by sedatives can be minimized, while facilitating achievement of an ideal sedative level. Additionally, the operating experience can be improved. The SAS can be effectively applied for preoperative assessment of sedation in pediatric bronchoscopy.

## Acknowledgments

The authors thank all participants for their involvement. The authors also thank Yuanyuan Liu for statistical consultation.

## Author contributions

Lin Zhong and Kun Shen are co-first authors and were responsible for data collection, data processing, statistical analysis, writing, and revision of the manuscript. Li Qiu is corresponding author and was responsible for study design and writing and revision of the manuscript. Ting Chen, Songhui Zhai, Qingfen Tao, Lina Chen, and Yuhong Tao were responsible for data collection, data processing, and statistical analysis.

**Conceptualization:** Lin Zhong, Kun Shen, Songhui Zhai, Qingfen Tao, Li Qiu.

**Data curation:** Lin Zhong, Kun Shen, Ting Chen, Qingfen Tao, Li Qiu.

**Formal analysis:** Lin Zhong, Kun Shen, Ting Chen.

**Funding acquisition:** Lin Zhong, Li Qiu.

**Investigation:** Lin Zhong, Songhui Zhai, Qingfen Tao, Lina Chen.

**Methodology:** Lin Zhong, Kun Shen, Songhui Zhai, Lina Chen, Li Qiu.

**Project administration:** Lin Zhong, Lina Chen, Li Qiu.

**Resources:** Lin Zhong, Ting Chen.

**Software:** Lin Zhong, Kun Shen, Ting Chen.

**Supervision:** Lina Chen, Yuhong Tao, Li Qiu.

**Validation:** Lina Chen, Li Qiu.

**Writing – original draft:** Lin Zhong, Kun Shen.

**Writing – review & editing:** Lin Zhong, Yuhong Tao, Li Qiu.

Lin Zhong orcid: 0000-0003-0587-3460.
